# Supplementary material for: EEG based assessment of stress in horses: a pilot study
Source: PeerJ. 2020 May 12;8:e8629. doi: 10.7717/peerj.8629 (PMC7227666; doi:10.7717/peerj.8629)
Supplement: Supplemental Information 1 [file peerj-08-8629-s001.docx]

| Table1: Significant differences regarding coupling coefficient of Canolty maps between rest and stress in horses. | | |
| --- | --- | --- |
| Phase of the slow wave/ [°] | Frequencies of the fast wave/ [Hz] | Corresponding p-values |
| 5.8 | 148.5, 154 | 0.0374, 0.0374 |
| 8.7 | 148.5, 154 | 0.0374, 0.0374 |
| 11.6 | 154 | 0.0374 |
| 14.5 | 154 | 0.0374 |
| 29 | 121 | 0.0374 |
| 31.9 | 121 | 0.0374 |
| 63.8 | 209 | 0.0374 |
| 66.7 | 198, 209 | 0.0374, 0.0163 |
| 69.6 | 198, 203.5, 209 | 0.0250, 0.0374, 0.0104 |
| 72.5 | 198, 203.5, 209 | 0.0374, 0.0104, 0.0065 |
| 75.4 | 198, 203.5, 209, 214.5 | 0.0374, 0.0104, 0.0065, 0.0163 |
| 78.3 | 198, 203.5, 209, 214.5 | 0.0374, 0.0065, 0.0065, 0.0104 |
| 81.2 | 198, 203.5, 209, 214.5, 220 | 0.0250, 0.0039, 0.0065, 0.0104, 0.0374 |
| 84.1 | 203.5, 209, 214.5 | 0.0039, 0.0065, 0.0250 |
| 87 | 203.5, 209, 214.5, 220 | 0.0039, 0.0065, 0.0374, 0.0374 |
| 89.9 | 203.5, 209, 214.5, 220 | 0.0250, 0.0065, 0.0374, 0.0374 |
| 92.8 | 209, 214.5, 220 | 0.0104, 0.0374, 0.0374 |
| 95.7 | 214.5, 220 | 0.0374, 0.0374 |
| 98.6 | 214.5, 220 | 0.0374, 0.0374 |
| 101.5 | 214.5, 220 | 0.0374, 0.0250 |
| 104.4 | 214.5, 220 | 0.0374, 0.0250 |
| 107.3 | 214.5, 220 | 0.0374, 0.0250 |
| 110.2 | 214.5, 220 | 0.0374, 0.0374 |
| 113.1 | 214.5, 220 | 0.0374, 0.0250 |
| 116 | 214.5, 220 | 0.0374, 0.0374 |
| 118.9 | 214.5, 220 | 0.0374, 0.0374 |
| 121.8 | 214.5, 220 | 0.0374, 0.0250 |
| 124.7 | 214.5, 220 | 0.0374, 0.0250 |
| 127.6 | 214.5, 220 | 0.0374, 0.0250 |
| 130.5 | 214.5, 220 | 0.0250, 0.0250 |
| 133.4 | 214.5, 220 | 0.0250, 0.0250 |
| 136.3 | 214.5, 220 | 0.0374, 0.0374 |
| 139.2 | 214.5, 220 | 0.0374, 0.0374 |
| 142.1 | 214.5, 220 | 0.0374, 0.0374 |
| 145 | 220 | 0.0374 |
| 197.2 | 225.5 | 0.0374 |
| 200.1 | 225.5 | 0.0374 |
| 203 | 225.5 | 0.0250 |
| 205.9 | 225.5 | 0.0374 |
| 208.8 | 225.5 | 0.0250 |
| 211.7 | 225.5 | 0.0250 |
| 214.6 | 187, 220, 225.5 | 0.0163, 0.0374, 0.0250 |
| 217.5 | 181.5, 187, 220, 225.5 | 0.0374, 0.0065, 0.0374, 0.0250 |
| 220.4 | 181.5, 187, 192.5, 220, 225.5 | 0.0104, 0.0065, 0.0374, 0.0374, 0.0250 |
| 223.3 | 181.5, 187, 192.5, 220, 225.5 | 0.0104, 0.0065, 0.0250, 0.0374, 0.0250 |
| 226.2 | 181.5, 187, 192.5, 225.5 | 0.0065, 0.0039, 0.0163, 0.0250 |
| 229.1 | 181.5, 187, 192.5, 225.5 | 0.0065, 0.0039, 0.0163, 0.0250 |
| 232 | 176, 181.5, 187, 192.5, 225.5 | 0.0250, 0.0065, 0.0039, 0.0163, 0.0250 |
| 234.9 | 165, 176, 181.5, 187, 192.5, 225.5 | 0.0374, 0.0250, 0.0039, 0.0039, 0.0163, 0.0374 |
| 237.8 | 165, 176, 181.5, 187, 192.5, 225.5 | 0.0374, 0.0163, 0.0039, 0.0039, 0.0163, 0.0374 |
| 240.7 | 165, 176, 181.5, 187, 192.5, 225.5 | 0.0374, 0.0140, 0.0039, 0.0039, 0.0250, 0.0374 |
| 243.6 | 176, 181.5, 187, 192.5, 198 | 0.0065, 0.0039, 0.0039, 0.0163, 0.0374 |
| 246.5 | 176, 181.5, 187, 192.5, 198, 225.5 | 0.0065, 0.0039, 0.0039, 0.0163, 0.0374, 0.0374 |
| 249.4 | 176, 181.5, 187, 192.5, 198, 225.5 | 0.0039, 0.0039, 0.0039, 0.0104, 0.0250, 0.0374 |
| 252.3 | 55, 176, 181.5, 187, 192.5, 198, 225.5 | 0.0374, 0.0039, 0.0039, 0.0039, 0.0104, 0.0163, 0.0250 |
| 255.2 | 55, 176, 181.5, 187, 192.5, 198, 220, 225.5 | 0.0250, 0.0039, 0.0039, 0.0039, 0.0104, 0.0163, 0.0374, 0.0163 |
| 258.1 | 55, 170.5, 176, 181.5, 187, 192.5, 198, 203.5, 220, 225.5 | 0.0250, 0.0039, 0.0039, 0.0039, 0.0104, 0.0163, 0.0374, 0.0250, 0.0250 |
| 261 | 55, 170.5, 176, 181.5, 187, 192.5, 198, 203.5, 220, 225.5 | 0.0250, 0.0374, 0.0039, 0.0039, 0.0039, 0.0104, 0.0163, 0.0374, 0.0250, 0.0250 |
| 263.9 | 55, 60.5, 165, 170.5, 176, 181.5, 187, 192.5, 198, 203.5, 225.5 | 0.0374, 0.0374, 0.0374, 0.0250, 0.0039, 0.0065, 0.0104, 0.0163, 0.0250, 0.0374, 0.0250 |
| 266.8 | 55, 60.5, 165, 170.5, 176, 181.5, 187, 192.5, 198, 203.5, 225.5 | 0.0374, 0.0374, 0.0374, 0.0104, 0.0039, 0.0065, 0.0104, 0.0250, 0.0250, 0.0374, 0.0374 |
| 269.7 | 55, 60.5, 99, 159.5, 165, 170.5, 176, 181.5, 187, 192.5, 198, 203.5, 225.5 | 0.0374, 0.0374, 0.0374, 0.0374, 0.0374, 0.0104, 0.0065, 0.0104, , 0.0104, 0.0163, 0.0250, 0.0374, 0.0374 |
| 272.6 | 55, 60.5, 99, 159.5, 170.5, 176, 181.5, 187, 192.5, 203.5 | 0.0374, 0.0374, 0.0374, 0.0374, 0.0104, 0.0065, 0.0104, 0.0104, 0.0374, 0.0374 |
| 275.5 | 55, 60.5, 99, 170.5, 176, 181.5, 187, 203.5 | 0.0374, 0.0374, 0.0374, 0.0104, 0.0104, 0.0163, 0.0250, 0.0374 |
| 278.4 | 55, 60.5, 99, 165, 170.5, 176, 181.5, 187, 203.5 | 0.0374, 0.0374, 0.0374, 0.0374, 0.0104, 0.0104, 0.0250, 0.0374, 0.0250 |
| 281.3 | 55, 60.5, 165, 170.5, 176, 181.5, 203.5, 214.5 | 0.0374, 0.0374, 0.0250, 0.0163, 0.0104, 0.0250, 0.0250, 0.0374 |
| 284.2 | 55, 60.5, 165, 170.5, 176, 181.5, 203.5, 209, 214.5 | 0.0374, 0.0374, 0.0374, 0.0163, 0.0104, 0.0250, 0.0250, 0.0374, 0.0250 |
| 287.1 | 55, 60.5, 176, 203.5, 209, 214.5 | 0.0374, 0.0374, 0.0250, 0.0163, 0.0374, 0.0250 |
| 290 | 60.5, 176, 203.5, 209, 214.5 | 0.0374, 0.0250, 0.0163, 0.0250, 0.0250 |
| 292.9 | 60.5, 176, 203.5, 209, 214.5 | 0.0374, 0.0374, 0.0250, 0.0163, 0.0163 |
| 295.8 | 203.5, 209, 214.5 | 0.0374, 0.0163, 0.0163 |
| 298.7 | 203.5, 209, 214.5,220 | 0.0374, 0.0104, 0.0104, 0.0374 |
| 301.6 | 203.5, 209, 214.5,220 | 0.0374, 0.0104, 0.0250, 0.0374 |
| 304.5 | 55, 203.5, 209, 214.5,220 | 0.0374, 0.0374, 0.0104, 0.0250, 0.0374 |
| 307.4 | 55, 209, 214.5,220 | 0.0374, 0.0104, 0.0250, 0.0374 |
| 310.3 | 209, 214.5,220 | 0.0104, 0.0163, 0.0374 |
| 313.2 | 209, 214.5,220 | 0.0104, 0.0163, 0.0250 |
| 316.1 | 209, 214.5,220 | 0.0104, 0.0163, 0.0374 |
| 319 | 209, 214.5 | 0.0104, 0.0163 |
| 321.9 | 209, 214.5 | 0.0163, 0.0250 |
| 324.8 | 71.5, 209, 214.5 | 0.0374, 0.0250, 0.0250 |
| 327.7 | 209, 214.5 | 0.0250, 0.0374 |
| 330.6 | 209, 214.5 | 0.0250, 0.0374 |
| 333.5 | 209 | 0.0250 |
| 336.4 | 209 | 0.0374 |
